# The Food-Contaminant Deoxynivalenol Modifies Eating by Targeting Anorexigenic Neurocircuitry

**DOI:** 10.1371/journal.pone.0026134

**Published:** 2011-10-12

**Authors:** Clémence Girardet, Marion S. Bonnet, Rajae Jdir, Medhi Sadoud, Sylvie Thirion, Catherine Tardivel, Julien Roux, Bruno Lebrun, Nicolas Wanaverbecq, Lourdes Mounien, Jérôme Trouslard, André Jean, Michel Dallaporta, Jean-Denis Troadec

**Affiliations:** 1 Université Paul Cézanne, Marseille, France; 2 INRA USC 2027, Marseille, France; 3 CNRS UMR 6231, Centre de Recherche en Neurobiologie-Neurophysiologie de Marseille, Département de Physiologie Neurovégétative, Marseille, France; 4 Université de la Méditerranée, Marseille, France; 5 Biomeostasis, Contract Research Organization, Marseille, France; Sapienza University of Rome, Italy

## Abstract

Physiological regulations of energy balance and body weight imply highly adaptive mechanisms which match caloric intake to caloric expenditure. In the central nervous system, the regulation of appetite relies on complex neurocircuitry which disturbance may alter energy balance and result in anorexia or obesity. Deoxynivalenol (DON), a trichothecene, is one of the most abundant mycotoxins found on contaminated cereals and its stability during processing and cooking explains its widespread presence in human food. DON has been implicated in acute and chronic illnesses in both humans and farm animals including weight loss. Here, we provide the first demonstration that DON reduced feeding behavior and modified satiation and satiety by interfering with central neuronal networks dedicated to food intake regulation. Moreover, our results strongly suggest that during intoxication, DON reaches the brain where it modifies anorexigenic balance. In view of the widespread human exposure to DON, the present results may lead to reconsider the potential consequences of chronic DON consumption on human eating disorders.

## Introduction

The capacity to adjust food intake in response to changing energy requirements is essential for survival. Recent progress has provided an insight into the central regulation of energy balance that links changes of body fat stores to adaptive adjustments of feeding behavior [1]. In the central nervous system (CNS), the regulation of appetite relies on complex neurocircuitry. Discrete neuronal pathways within specific brain areas, mainly the hypothalamus and the brainstem, are clearly involved in this control of feeding behavior. Peripheral information linked to fat deposit or nutriment availability are implicated as endogenous signaling molecules in the control of energy expenditure, and initiation and termination of a meal. The primary targets of these peripheral molecules are first-order anorexigenic and orexigenic neurons that express pro-opiomelanocortin (POMC)/cocaine- and amphetamine-regulated transcript (CART) and neuropeptide Y (NPY)/Agouti-related peptide (AgRP) respectively. The physiological importance of this homeostatic control system is highlighted by the severe eating disorders (obesity, anorexia, cachexia) that result from the dysfunction or any of several of its key components.

Deoxynivalenol (DON), also commonly called vomitoxin, is a trichothecene mycotoxin mainly produced by *Fusarium* fungi. DON is one of the most abundant trichothecenes found on cereals such as wheat, barley, oats, rye, and maize, and less often in rice grown in Europe, America and Asia [2]. The extent of cereal contamination is strongly associated with rainfall and moisture at the time of flowering and with grain storage conditions. DON has been implicated in mycotoxicoses in both humans and farm animals. High doses toxicity of DON is characterized by a set of symptoms including diarrhea, vomiting, leukocytosis, hemorrhage, circulatory shock and death whereas low doses toxicity is characterized by anorexia, reduced weight gain, diminished nutritional efficiency, neuroendocrine changes and immunologic effects [2]. In farm animals including poultry and ruminants, intoxication following consumption of cereals and cereal-derived products contaminated with DON results in feed refusal and reduced weight gain. These symptoms lead to growth retardation and can have great economic consequences. In humans, epidemiological studies have reported acute illnesses including vomiting, abdominal pain, diarrhea, headache, dizziness in populations who have consumed *Fusarium*-contaminated grains and in addition, DON is suspected to increased occurrence of more severe diseases such as esophageal cancer [3]. The levels and patterns of human food commodities contamination justify that DON consumption constitutes a major issue for global public health. A survey by Lombeart and colleagues reported the detection of DON in 63% of 363 cereal-based infant foods [4]. Similarly, a European food study carried out in 12 countries revealed that, out of a total of 11.022 samples analyzed, more than a half contained DON [5]. DON stability during processing and cooking explains its widespread presence in human food. Trichothecenes, including DON, are actually stable at 120°C, moderately stable at 180°C, decompose within 30–40 min at 210°C [6], and autoclave processing reduces by only 12% maize contamination [7]. In view of this widespread human exposure to DON, studies improving our knowledge of DON toxicity remain essential and should be conducted. While the peripheral action of DON was extensively studied [1], data illustrating its effects on CNS are significantly less abundant. Despite the described modulation of feeding behavior induced by DON consumption, the data aiming to characterize this effect are limited and the mechanisms by which this toxin exerts its action remain largely unknown. Accordingly, additional information are strongly needed to more accurately understand how such a commonly encountered toxin can so severely modify animal and human food intake.

Using a multidisciplinary approach, we accessed to mechanisms underlying anorexia induced by acute DON intoxication. In conclusion, we showed that, in addition to a peripheral action, the toxin having reached the brain after *per os* administration can act centrally and results in the impairment of anorexigenic/orexigenic balance. These data may lead to reconsider the consequence of the chronic consumption of low DON doses on the development of pathophysiological alteration of food intake behavior.

## Results

### 1- Acute *per os* administration of DON alters night-time food intake and meal microstructure

**Figure 1:**
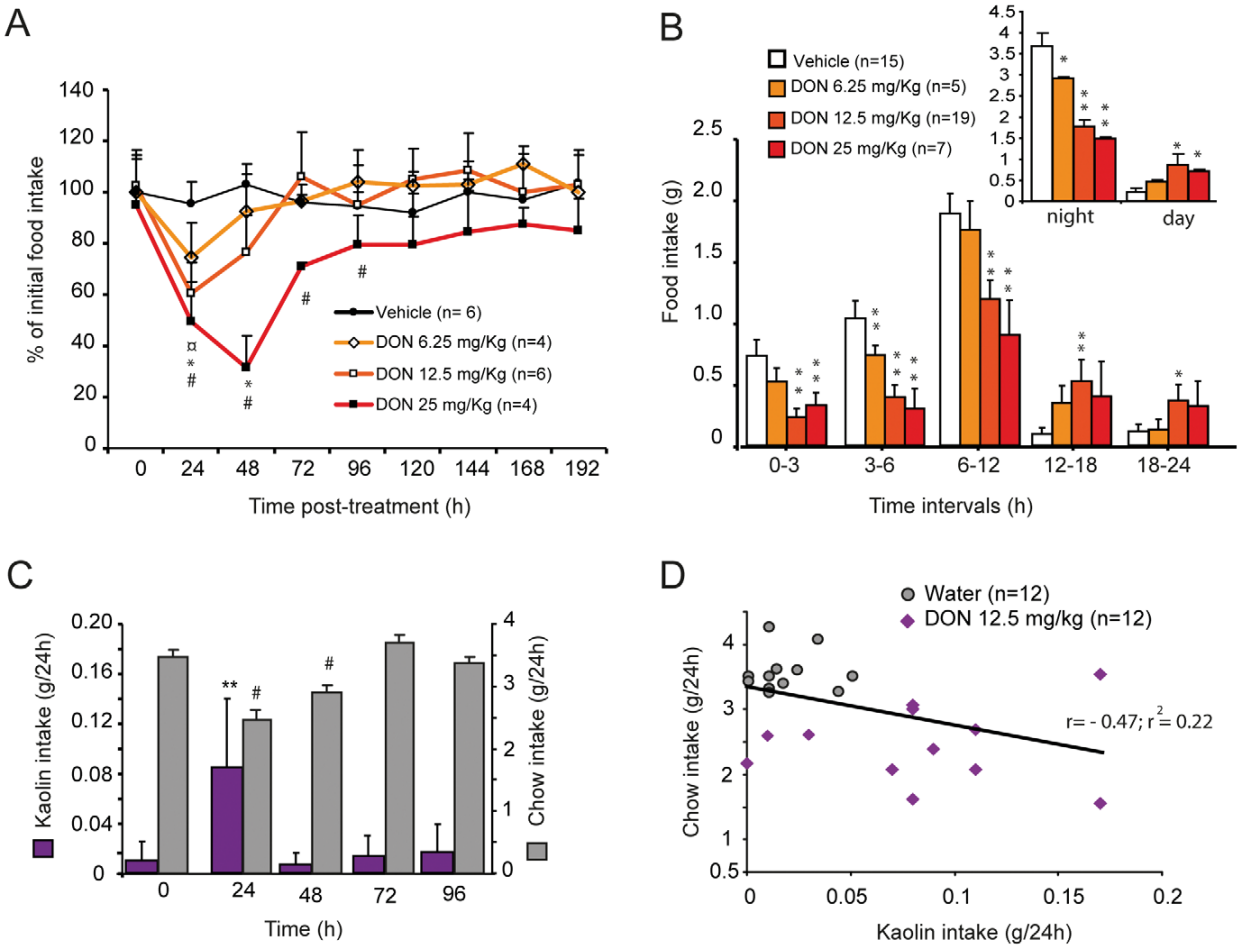
Acute *per os* DON administration modifies night-time food intake. **A:** Daily food intake (% of initial food intake) measured from 24 to 192 h after oral gavage of either water (vehicle) or DON (6.25, 12.5 and 25 mg/kg) in adult mice. **B:** Food intake (g), measured over the first 24 h period, of mice having received an oral gavage of either water or DON (6.25, 12.5 and 25 mg/kg). **C:** Kaolin intake and regular chow intake measured 0, 24, 48, 72 and 96 h after DON (12.5 mg/kg) administration. **D:** Correlation of kaolin intake and chow intake by mice that received an oral gavage of either water or DON (12.5 mg/kg). In A: ¤; *; # represent *P*<0.05, significantly different from vehicle-treated mice, respectively for 6.25, 12.5 and 25 mg/kg DON-treated mice. In B: *P<0.05 and **P<0.01, significantly different from vehicle-treated mice. In C: **P<0.01, significantly different from control Kaolin intake. # *P*<0.05, significantly different from control chow intake.

**Figure 2:**
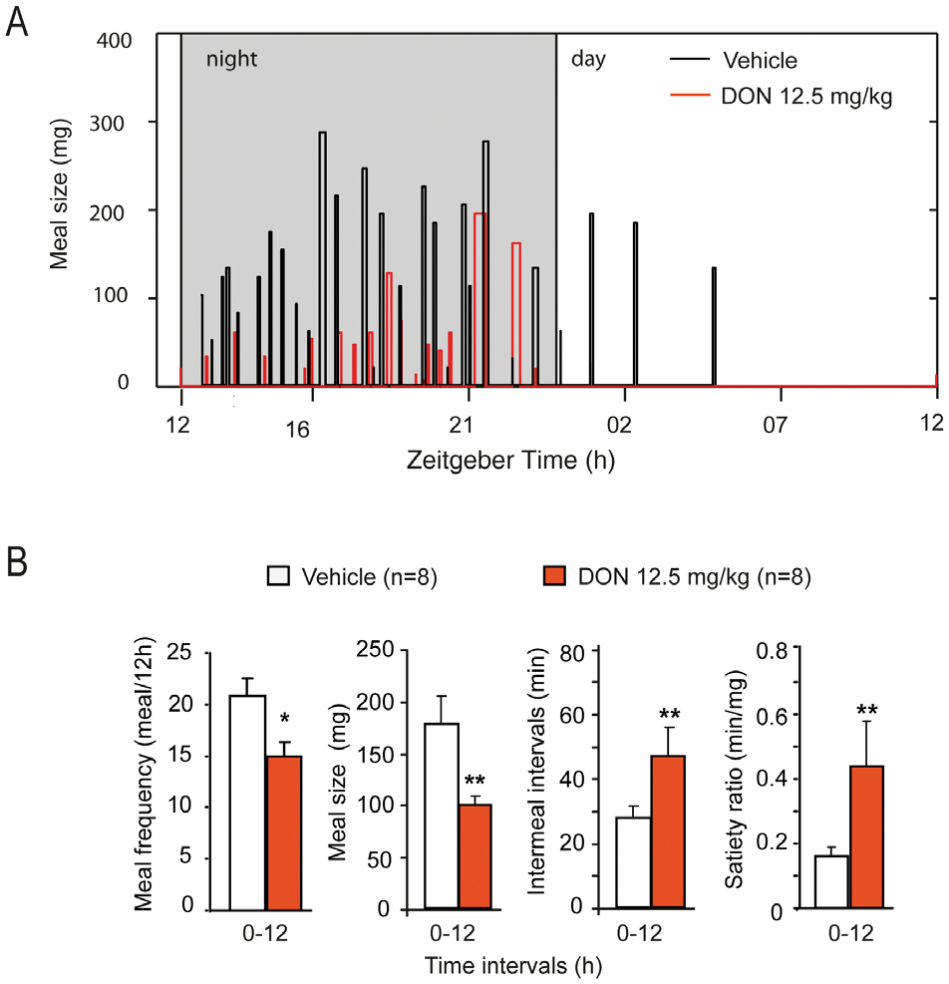
Effect of acute *per os* DON administration on meal microstructure. **A:** Graph showing meal size over 24 h after an oral gavage of either water (vehicle, black histograms) or DON (12.5 mg/kg, red histograms) in an adult mouse. Note that the width of each bar represents the meal duration. Time is indicated in zeitgeber time, 12 being the transition between lights on and lights off and 0 or 24 the transition between lights off and lights on. The dark period is represented by the shaded box. **B:** Meal microstructure analysis performed during the dark period for mice that received either water (white bars) or DON (12.5 mg/kg, orange bars). *P<0.05 and **P<0.01, significantly different from vehicle-treated mice.

A single oral administration of DON resulted in a dose-dependent decrease in daily food intake with a notably long-lasting effect for the highest doses (Figure 1A). Note that 6.25, 12.5 and 25 mg/kg of DON diminished respectively by 24, 39 and 47% food intake measured during the first 24 h following administration. Food consumption measured 3, 6, 12 and 18 h after treatment revealed that DON profoundly affected the night-time food intake (Figure 1B). To decipher feeding behavior analysis during DON intoxication, we quantified the consumption of a non-nutritive substance i.e. kaolin. This behavior, known as pica, serves as a model for the study of nausea/emesis in rodents [8]. While mice treated with vehicle consumed 18.3+/−4.8 mg/24 h of kaolin (time 0 on Figure 1C), 12.5 mg/kg of DON caused a significant increase in kaolin intake (83.3+/−16.2 mg/24 h; P<0.01). This behavior was not observable any more 48 h post-injection, while anorexia was still ongoing. In the DON treated-mice, daily standard chow and kaolin intakes were not significantly correlated (*r*-value:0.1). Plotting the standard chow and kaolin intakes for the vehicle- and DON-treated animals on the same graph revealed that these two parameters were poorly correlated (*r*-value:−0.47, r^2^:0.22; Figure 1D). Using continuous recording of food consumption (Figure 2A), we next analyzed the effect of DON (12.5 mg/kg) administration on meal parameters computed for each animal over all meals during the trial period. This monitoring confirmed that DON essentially modified food intake during the dark phase (Figure 2A). Given the modification of nightly food intake observed in response to DON, meal microstructure analysis was then computed for the dark phase (0–12 h). Compared with vehicle, DON reduced meal frequency by 27.9% (15.0+/−1.3 *versus* 20.8+/−1.7 meals/12 h, P<0.05) and meal size by 44.2%(99.4+/−8.4 mg *versus* 178.1+/−26.8 mg, P<0.01) and increased intermeal intervals by 68%(47.5+/−8.9 min *versus* 28.2+/−3.5 min, P<0.01). During this trial period, the satiety ratio was also increased by 40% in response to the toxin (P<0.01; Figure 2B).

### 2- Brain pattern of central pathways activated in response to DON *per os* administration

**Figure 3:**
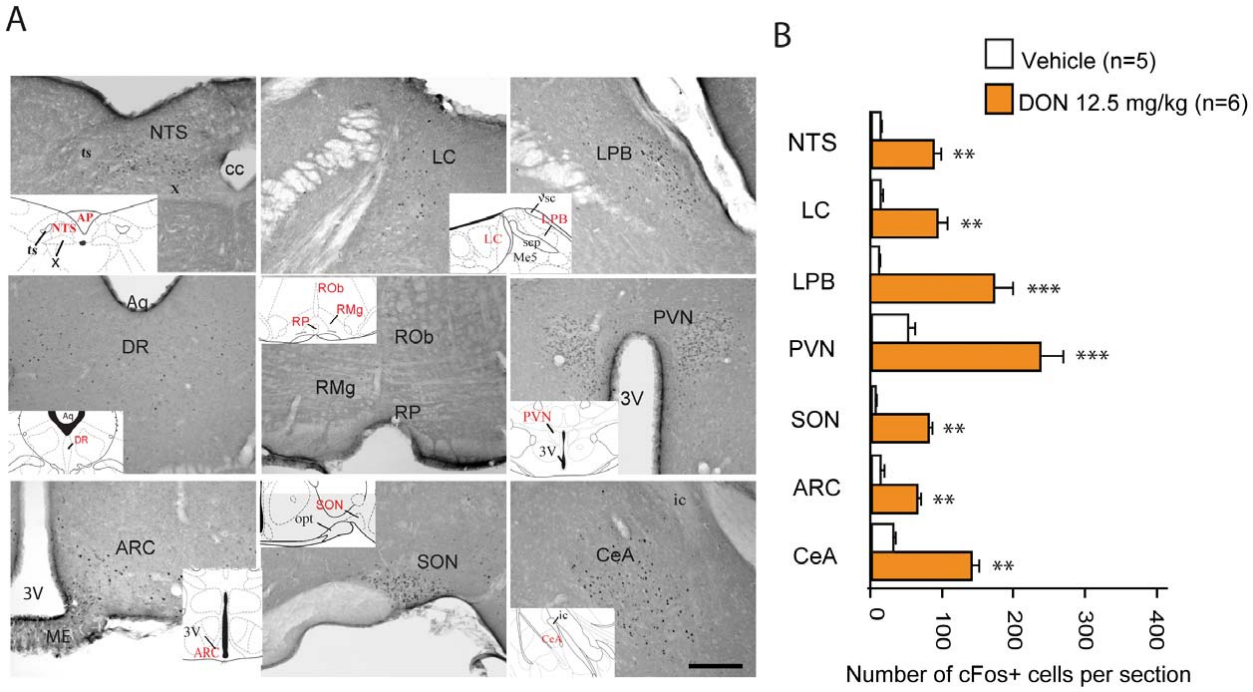
Effects of *per os* DON administration on c-Fos immunoreactivity. **A:** Representative coronal sections illustrating the c-Fos labeling observed within brainstem, pons and forebrain regions of mice treated with DON (12.5 mg/kg) and sacrificed 3 h post-treatment. Inserts show the level and the location where the photomicrographs originate from **B:** Quantification of the number of c-Fos immunoreactive nuclei within brainstem, pons and forebrain nuclei observed in mice treated either with vehicle (water, white bars) or DON (12.5 mg/kg, orange bars). *P<0.05, **P<0.01, ***P<0.001 significantly different from vehicle-treated mice. 3V, third ventricle, AP, area postrema; ARC, arcuate nucleus; Aq, Aqueduct; cc, central canal; CeA, central amygdala; DR, dorsal raphe; LPB, lateral parabrachial; LC, locus coeruleus; ic, insular cortex; Me5, mesencephalic trigeminal nucleus; NTS, nucleus tractus solitarius; opt, optic tract; PVN, paraventricular nucleus; RMg, raphe magnus; ROb, raphe obscursis; RP, raphe pallidus; scp, superior cerebellar peduncle; SON, supraoptic nucleus; X, dorsal motor nucleus of the vagus; ts, tractus solitarius; vsc, ventral spinocerebellar tract. Scale bar: 100 µm.

Central structures activated in response to *per os* administration of DON were next identified using the immune detection of the c-Fos protein. A very low basal level of c-Fos positive nuclei was observed in the brainstem, pons and forebrain of water-treated mice (Table 1). DON-treated mice exhibited a strong rise in the number of c-Fos positive nuclei within the nucleus tractus solitarius (NTS) and a moderate increase within the area postrema (AP, Figure 3 and Table 1) and ventrolateral medulla (VLM). Noticeably, the serotoninergic raphe formation did not exhibit any DON-induced c-Fos expression whatever the rostro-caudal level considered (Figure 3 and Table 1). Animals challenged with DON also displayed a strong rise in c-Fos immunoreactivity in the lateral part of the parabrachial nucleus (LPB) and in the locus coeruleus (LC), and a moderate-to-strong c-Fos staining within forebrain structures such as the paraventricular hypothalamus nucleus (PVN), arcuate nucleus (ARC), median eminence (ME), bed nucleus of the stria terminalis (BST) and central nucleus of the amygdala (CeA) (Figure 3; Table 1).

**Table 1:** c-Fos signal in brain structures of vehicle or DON-challenged mice.

	*per os* vehicle (water)	*per os* DON (12,5 mg/kg)	icv vehicle (NaCl)	icv DON (12,5 mg/kg)
**Diencephalon**				
Subfornical organ	0/+	**++**	+	**++**
Paraventricular thalamic nuclei	+++	+++	++	++
Central nucleus of the amygdala	0/+	**+++**	0/+	**+**
Laterodorsal part of the bed of the stria terminalis	0/+	**+++**	0/+	**+**
*Hypothalamus*				
Preoptic area	0/+	**+/++**	+	**+++**
Anterior area	+	**++**	+	**++**
Paraventricular nucleus	0/+	**+++**	+	**+++**
Supraoptic nucleus	+	**+++**	0/+	**++**
Median eminence	0/+	**++**	+	+
Dorsomedian nucleus	0/+	0/+	0/+	**+**
Ventromedian nucleus	0/+	0/+	0/+	0/+
Lateral area	+	**++**	+	**++**
Posterior area	+	**++**	+	**++**
Parasubthalamic nucleus	0/+	**+++**	0/+	**++**
Arcuate nucleus	0/+	**++**	0/+	**+++**
**Brainstem**				
Dorsal raphe nucleus	0/+	0/+	0/+	0/+
Laterodorsal tegmental nucleus	+	**++**	+	**++**
Parabrachial nucleus	0/+	**+++**	0/+	**+++**
Raphe magnus nucleus	0/+	0/+	0/+	0/+
Locus coeruleus	+	**+++**	0/+	**+++**
Motor trigeminal nucleus	0/+	**++**	0/+	0/+
Nucleus of trapezoid body	+++	+++	0/+	0/+
Area postrema	0/+	**+/++**	0/+	**+**
Caudal NTS	0/+	**++**	0/+	**++**
Postremal NTS	0/+	**+++**	0/+	**+++**
Rostral NTS	0/+	**+++**	0/+	**+++**
Dorsal motor nucleus of the vagus	0/+	**+**	0/+	**++**
Raphe obscursus nucleus	0/+	0/+	0/+	0/+
Raphe pallidus nucleus	0/+	0/+	0/+	0/+
Caudal Ventolateral medulla	+	**++**	0/+	**++**
Rostral ventolateral medulla	+	**++**	0/+	**++**
Spinal nucleus trigeminal	0/+	0/+	0/+	0/+
**Other**				
Ependymal cells	0/+	0/+	0/+	**++**
Choroïd plexus	0/+	**++**	+	**++**

0 = no stained cell; + = 1−30 cells per section; ++ = 31−60 cells; +++ = 61 and more cells. Bold indicates brain structures where c-Fos signal is significantly different between vehicle and DON-treated mice.

### 3- Phenotypic characterization of DON-activated neurons

**Figure 4:**
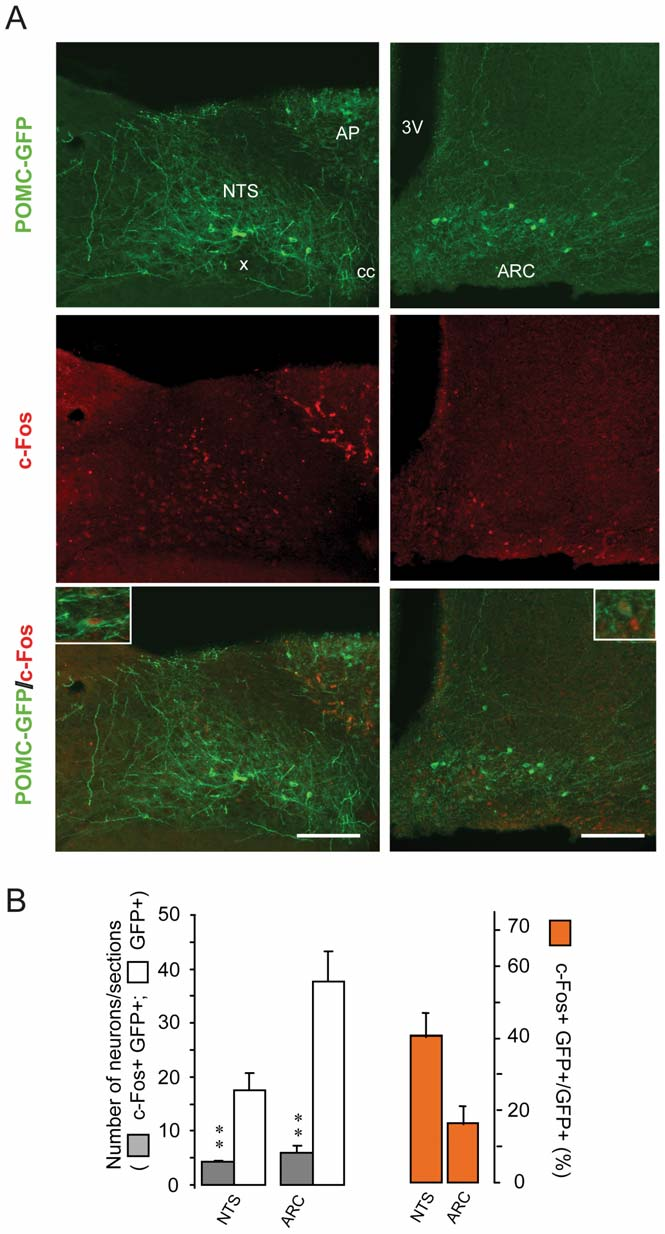
Impact of *per os* DON administration on POMC neurons activation. **A:** Representative photomicrographs of POMC-GFP (green) and c-Fos (red) double labeling performed on hypothalamic and brainstem coronal sections of mice treated with DON. Inserts show high magnification images of double-labeled neurons. **B:** Quantification of the number of POMC-GFP positive neurons (white bars) and c-Fos double-labeled cells (gray bars) in the NTS and ARC after DON treatment. The orange bars represent the percentage of double-labeled neurons in each area. All immunostaining were obtained from animals sacrificed 3 h after a 12.5 mg/kg DON administration. 3V, third ventricle; AP, area postrema; ARC, arcuate nucleus; cc, central canal; NTS, nucleus of the solitary tract; X, dorsal motor nucleus of the vagus. Scale bars: 150 µm. **P<0.01, significantly different from vehicle-treated mice. ns, non significantly different from vehicle-treated mice.

**Figure 5:**
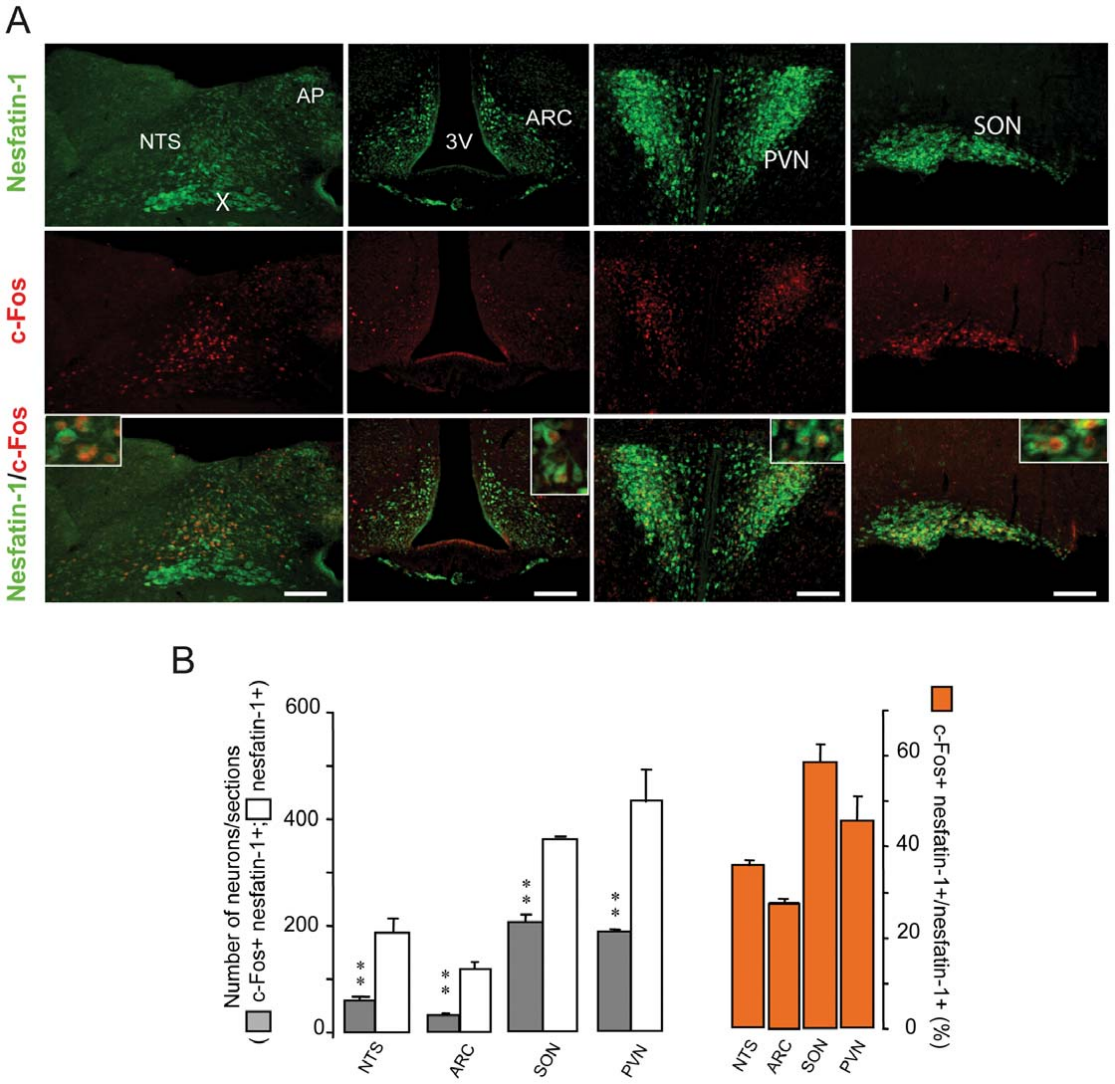
Nesfatin-1 and c-Fos double immunochemistry performed on DON-treated mice. **A:** Co-detection of nesfatin-1 expressing neurons (green) and c-Fos (red) positive cells on brainstem and hypothalamic coronal sections of mice treated with DON. Note the presence of double-labeled neurons (inset). **B:** Quantification of the number of nesfatin-1-positive neurons (white bars) and c-Fos double-labeled cells (gray bars) in the NTS, ARC, PNV and SON after DON treatment. The orange bars represent the percentage of double labeled neurons per phenotype in each area. All immunostaining were obtained from animals sacrificed 3 h after a 12.5 mg/kg DON administration. Inserts show high magnification images of double labeled neurons. 3V, third ventricle; AP, area postrema; ARC, arcuate nucleus; cc, central canal; PVN, paraventricular nucleus; NTS, nucleus of the solitary tract; SON, supraoptic nucleus; TH, tyrosine hydroxylase; X, dorsal motor nucleus of the vagus. Scale bars: 200 µm. **P<0.01, significantly different from vehicle-treated mice. ns, non significantly different from vehicle-treated mice.

**Figure 6:**
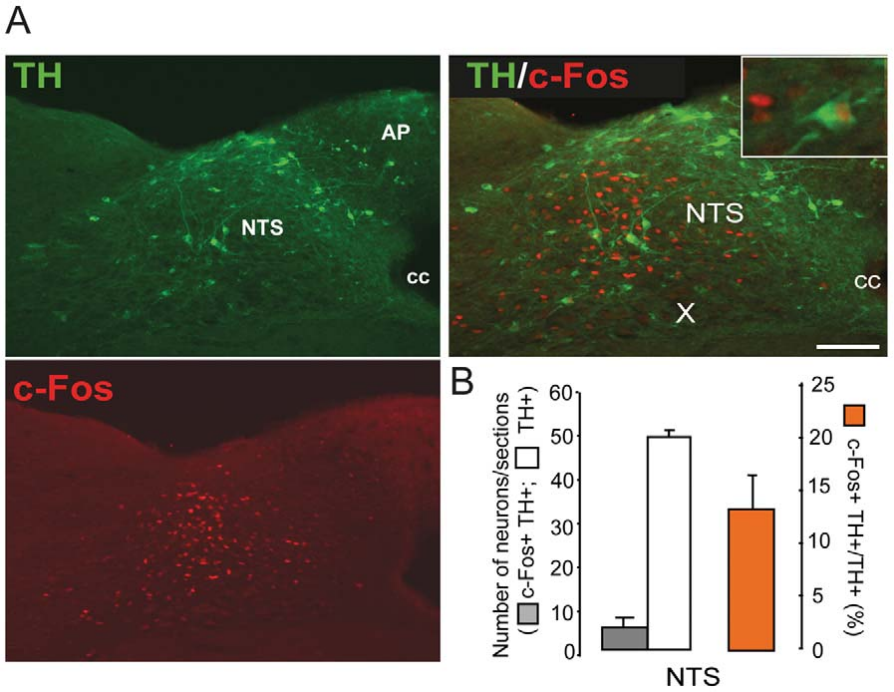
Effect of *per os* DON administration on brainstem A2/C2 neurons activation. **A:** Visualization of TH-expressing neurons (green) and c-Fos (red) positive cells on brainstem coronal sections of mice treated with DON. Inserts show high magnification images of double-labeled neurons. **B:** Quantification of the number of TH positive neurons (white bars) and c-Fos double-labeled cells (gray bars) in the NTS after DON treatment. The orange bars represent the percentage of double labeled neurons per phenotype in each area. All immunostaining were obtained from animals sacrificed 3 h after a 12.5 mg/kg DON administration. AP, area postrema; cc, central canal; NTS, nucleus of the solitary tract; TH, tyrosine hydroxylase; X, dorsal motor nucleus of the vagus. Scale bars: 100 µm. **P<0.01, significantly different from vehicle-treated mice. ns, non significantly different from vehicle-treated mice.

**Figure 7:**
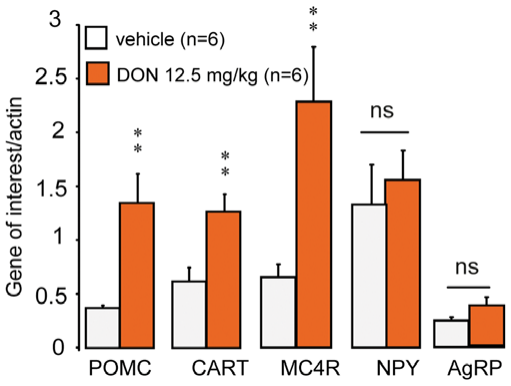
Effects of *per os* DON administration on hypothalamic mRNA expression. Quantification of POMC, CART, MC4R, NPY and AgRP transcript expression within the hypothalamus of water- (white bars) or DON-treated mice (12.5 mg/kg, orange bars). **P<0.01, significantly different from vehicle-treated mice. ns, non significantly different from vehicle-treated mice.

The use of POMC-GFP transgenic mice revealed that oral DON administration (12.5 mg/kg) significantly increased the number of POMC neurons positive for c-Fos, both in the NTS and in the ARC (Figure 4). Interestingly, more than 40% of POMC neurons were found immunoreactive for c-Fos within the NTS (Figure 4B). Similarly, the number of nesfatin-1 neurons labeled for c-Fos in the NTS and hypothalamic nuclei (ARC, PVN, SON) was greatly increased by the treatment (Figure 5). For these structures, the percentage of double labeled neurons was comprised between 24 and 58% of the entire nesfatin-1 neuron population (Figure 5B). Finally, double immunostaining of c-Fos/Tyrosine Hydroxylase (TH) cells in the brainstem sections revealed that about 15% of catecholaminergic (TH-containing) neurons in the NTS were c-Fos immunoreactive after DON treatment (Figure 6). Note that control animals (water-treated) were virtually devoid of double labeled cells. Using real-time RT-PCR analysis on hypothalamic samples, we next quantified POMC, CART, melanocortin-4 receptor (MC4R), NPY and AgRP mRNA expression. We observed that POMC, CART and MC4R mRNA expression strongly increased following the treatment while NPY and AgRP mRNA remained unaffected (Figure 7).

### 4- Unilateral cervical vagotomy does not alter DON-induced c-Fos expression within the brainstem

**Figure 8:**
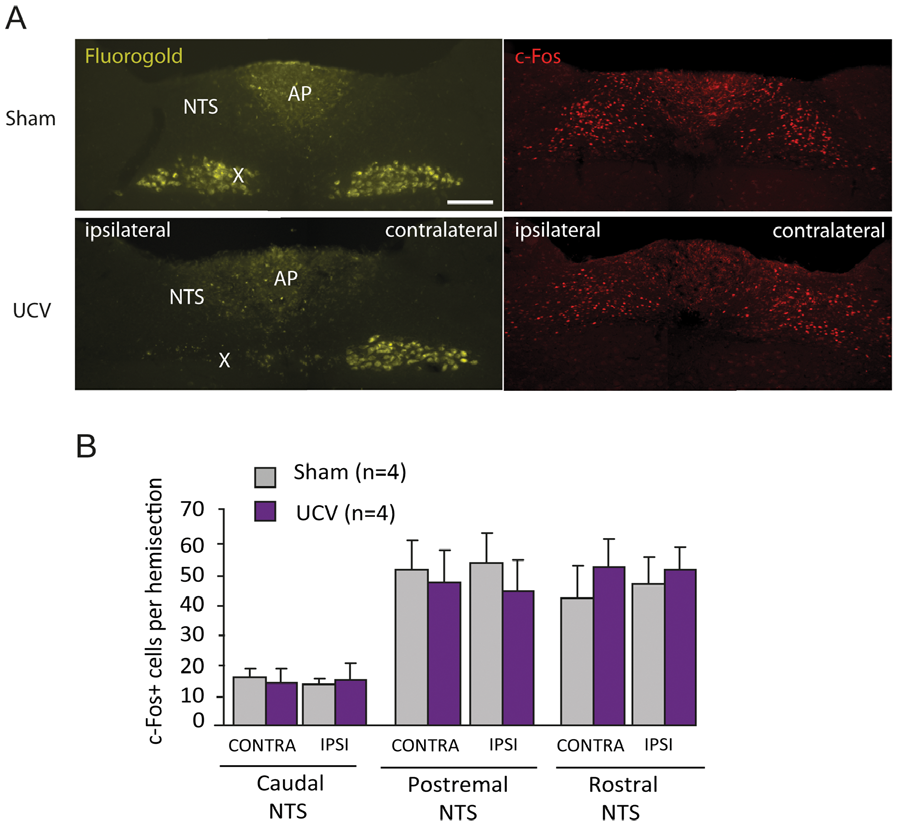
Effect on vagotomy on brainstem DON-induced c-Fos expression. **A:** Detection of fluorogold (yellow) and c-Fos (red) on brainstem coronal sections of sham or unilaterally vagotomized (UCV) mice after an oral administration of DON (12.5 mg/kg). Note that after vagotomy no fluorogold labeling can be visualized on the ipsilateral DMNX. **B:** Quantification of DON-induced (12.5 mg/kg) c-Fos immunoreactivity in subareas of the NTS in sham (grey bars) or vagotomized (purple bars) mice.

The contribution of the vagus nerve in the conveyance of DON signaling from the gastrointestinal tract toward the brainstem was evaluated by studying the impact of unilateral cervical vagotomy (UCV) on DON-induced c-Fos expression within the brainstem. Seven days before DON administration and sacrifice, animals were given an intraperitoneal injection of Fluorogold for verification of the lesion. The absence of the retrograde neuronal tracer fluorogold in the cell columns of the dorsal motor nucleus (DMNX) corresponding to the sectioned branch of the vagus attested of the correct lesion of the nerve (Figure 7A). c-Fos immunoreactivity within the NTS, consecutive to DON treatment, was not significantly altered by UCV. Furthermore, NTS ispsi- and contra-lateral sides did not differ significantly, whatever the rostro-caudal NTS level analyzed (P>0.05; Figure 8).

### 5- Intracerebroventricular (icv) DON injection on induced anorexia and neuronal activation

**Figure 9:**
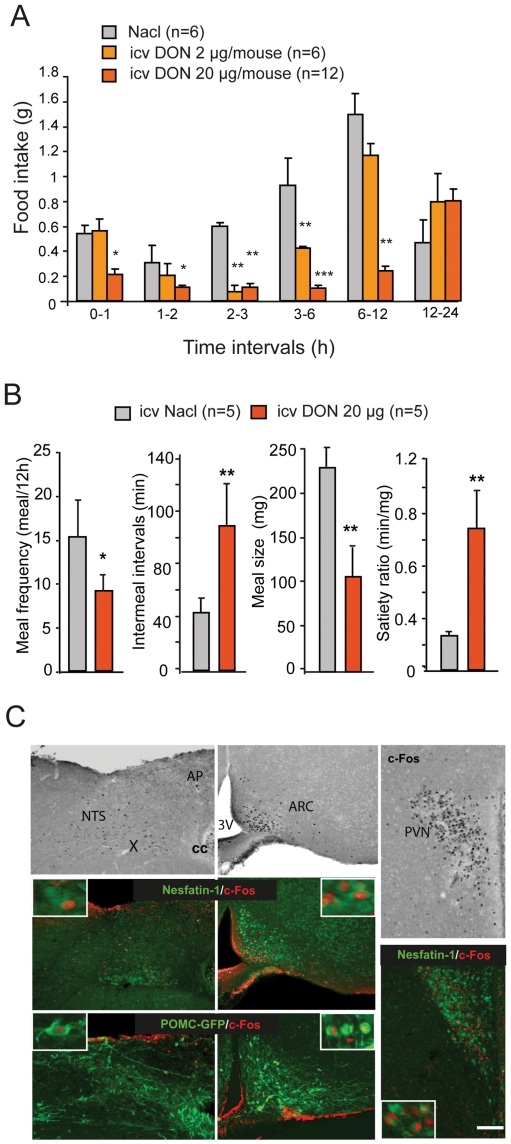
Effect of intracerebroventricular DON injection. **A:** Food intake of mice having received either an icv injection of saline (vehicle) or DON at different doses (2 or 20 µg/mouse). **B:** Effect of icv DON injection (20 µg/mouse) on meal parameters. **C:** c-Fos, nesfatin−1/c-Fos and POMC-GFP/c-Fos labeling on brainstem and hypothalamic coronal sections 1 h after an icv injection of DON (20 µg/mouse). Inserts in E show higher magnification images of double labeled neurons. 3V, third ventricle; AP, area postrema; ARC, arcuate nucleus; cc, central canal; NTS, nucleus of the solitary tract; X, dorsal motor nucleus of the vagus. Scale bars 100 µm *P<0.05, **P<0.01 and significantly different from vehicle-treated mice.

To address the possible direct central action of DON, we next performed icv injections (lateral ventricles) of the toxin on chronically cannulated mice. At doses ineffective at the periphery, central DON injections dose-dependently (2–20 µg/mouse) reduced food intake measured during the dark phase (Figure 9A). It should be noted that these DON doses are about 20–100 fold lower than previously used *per os* administered DON doses (equivalent to 125–500 µg/mouse). Interestingly, at the dose of 20 µg/mouse, DON reduced night-time food intake as soon as 1 h after injection and the effect was long-lasting during the following 12 h post-injection. Compared with vehicle, this dose decreased meal frequency by 40.1% (15.7+/−4.3 *versus* 9.4+/−1.8 meals/12 h, P<0.05) and meal size by 53.3% (233.0+/−19.7 mg *versus* 108.7+/−31.4 mg, P<0.01; Figure 9B). Finally, c-Fos analysis performed on animals centrally injected with the toxin revealed a pattern of labeled structures quite similar to that observed in animals having received a *per os* administration of the toxin (Figure 9C and Table 1). c-Fos labeling was indeed observed within the NTS, AP, LPB, LC and hypothalamic nuclei including ME, ARC and PVN. Central DON injection resulted also in a significant increase in c-Fos positive POMC and nesfatin-1 expressing neurons located both in the brainstem and hypothalamus (Figure 9C).

## Discussion

The mechanisms underlying the modulation of food intake behavior by DON and its central targets are until now unclear. To investigate these mechanisms, we performed acute DON administrations. We characterized DON intoxication by showing that the toxin affects feeding behavior by modulating both satiety and satiation. Using c-Fos expression mapping, we first identified that part of neuronal pathways activated by the toxin belong to prototypic anorexigenic pathways. Finally, by performing central injection, we demonstrated that DON could directly act on the brain to modify eating. This new results may lead to reconsider the impact of chronic consumption of this toxin on human health.

### Acute DON intoxication results in the alteration of satiety and satiation

While pig was reported to be the most sensitive species to DON, numerous *in vivo* and *in vitro* DON toxicity studies were conducted on rodents and especially on mice which can be considered as a good model for the study of DON toxicity [9]–[10] and DON-induced anorexia [11]. The doses of DON used here are classically employed to study acute intoxication in mice [12]–[13]. *Per os* intoxication induced a dose-dependent reduction in daily food intake by decreasing night-time food consumption. By performing meal pattern analysis, which allows the continuous study of *ad libitum* eating and provides a detailed description of the elements of eating behavior, we revealed that *per os* DON decreased both meal frequency (satiety) and size (satiation). The observed effect on meal frequency was evocative of nausea-induced anorexia [14]–[15]. On the other hand, the consequence of DON treatment on meal size suggested the action of DON on meal termination mechanisms. A reduction in meal size but not in meal frequency is classically observed with physiological regulators that potently inhibit food intake such as leptin [16], cholecystokinin [17], peptide YY 3–36 [18], insulin [19] or melanocortin agonists [20]. Moreover, the clay (kaolin) intake, used here as an indicator of nausea/emesis [8], was poorly correlated with standard chow intake. This suggesting that malaise and nausea did not totally explain anorexia. Hence, altogether our results revealed a complex action of DON on food intake where DON-induced anorexia depends not only on modification of satiety but also on satiation modulation.

### DON activates central anorexigenic pathways

To date, central structures activated in response to DON were not clearly identified. Using c-Fos staining which remains a useful approach to identify activated neuronal groups, we revealed the activation of key autonomic areas and hypothalamic nuclei. A significant elevation of c-Fos immunoreactivity was also observed in the central nucleus of the CeA and the dorsolateral division of the BST which are involved in the integration of emotional stimuli. This pattern is consistent with a coordinated autonomic, endocrine, and behavioral (anorexia) response to DON.

Regardless of sometimes conflicting results and evident interspecies differences, it was initially proposed that the toxin modifies serotoninergic and catecholaminergic activities in the CNS, thereby altering neurotransmission in brain structures such as the hypothalamus, frontal cortex, and cerebellum. Accordingly, this observed elevated brain serotonin turnover was supposed to be responsible for the vomiting effects of DON [1]. The capacity of serotonin receptor antagonists to prevent DON-induced emesis in swine supported this assumption [21]. Surprisingly, it should be noted that the serotoninergic raphe nuclei did not display any increased c-Fos labeling in response to DON, suggesting that on the contrary to previous assumption (see [2] for review), central serotonin release does not significantly contribute to DON-induced hypophagia.

Moreover, we report here that a significant proportion of the DON-activated neurons belong to central pathways strongly dedicated to the control of food intake. These cell groups include POMC- and nesfatin-1-expressing neurons located both in key hypothalamic nuclei and NTS. The demonstration of POMC-expressing neurons activation is crucial since these neurons are well recognized to intervene in normal energy homeostasis both in rodents and humans [22]. POMC neurons which co-express CART neurons relay the anorexigenic action of emblematic adiposity signals leptin or insulin. In these neurons, POMC is cleaved to α-melanocyte stimulating hormones (α-MSH) which acts on melanocortin 3 and melanocortin 4 receptors (MC3R and MC4R) of neurons mainly located in the PNV to reduce appetite. In accordance, endogenous and synthetic agonists for MC3R/MC4R have been shown to cause hypophagia and weight loss in experimental animals [23]. The catabolic action of both leptin and insulin relies upon α-MSH signaling since the central administration of MC3R/MC4R antagonists was shown to block their actions in the brain. Both insulin and leptin increase the activity of anorexigenic POMC/CART neurons in the ARC. POMC/CART neuronal population, also called “first-order neurons”, widely project to “second-order neurons” located in the PVN, the LHA, the DMH and to areas of the hindbrain involved in satiation and satiety control [24]–[25]. The POMC/CART-originating tract has an overall catabolic effect whose increased activity induces a decrease in food intake and increased energy expenditure. Similarly, Nesfatin–1, an 82 amino-acid peptide derived from the cleavage of the precursor NUCB2, was identified in 2006 by Oh-I and colleagues [26]. This peptide exerts potent anorexigenic action after either peripheral or central administration [26]. The interest generated by this peptide is based on its anorexigenic action performed independently of leptin signalization after injection in both the cerebral ventricles [26] and the peritoneal cavity [27]. Nesfatin-1 was found expressed in neurons of various brain areas including hypothalamic nuclei (PVN, ARC) and at the brainstem level (NTS, DMNX) [28]–[29]. Nesfatinergic neurons located in these structures are activated in response to refeeding or intraperitoneal injection of CCK suggesting that central nesfatin-1 could participate in the meal termination mechanisms [30]. Moreover, Nesfatin-1 has a food intake-reducing effect that is linked to the recruitment of the melanocortinergic pathway [31]. The observation of DON-induced POMC and nesfatin–1 expressing neurons activation suggest that the release of these anorexigenic signals and related compounds could partake in the feeding reducing action of the toxin. Strengthening these results, we observed that DON modulated mRNA levels of the hypothalamic melanocortinergic pathway including POMC, CART and MC4R, whereas mRNA of orexigenic effectors i.e. NPY and AgRP remained unaffected. Interestingly, POMC and CART mRNA expression is increased in response to leptin while they are reduced in fasted or obese (ob/ob or db/db) animals [32]. Hence, these results confirmed that in response to DON intoxication anorexigenic pathways are recruited. Finally, DON activated brainstem/hypothalamus connecting networks as attested by the activation of the A2/C2 catecholaminergic group. Altogether, these data strongly support the assumption that DON interferes directly or indirectly with neuronal networks devoted to central energy balance and that this action could partly explain the DON-induced hypophagia and the modification of meal frequency and size we observed in response to the toxin.

### DON can directly target the brain

While we reported the activation of central neurocircuitry in response to *per os* DON administration, the question is how does DON signal to the brain? The stimulation of the vagus nerve by DON itself or peripheral compounds released in response to the toxin could be assumed. To investigate whether *per os* DON administration and probable consecutive mucosal gastric irritation signal to the brain through vagal terminals innervating the gastrointestinal tract, we submitted mice to UCV. UCV, previously used to consider the contribution of the vagus nerve to c-Fos expression within the NTS in response to various conditions [33]–[34], failed to reduce DON-evoked c-Fos expression in the ipsilateral NTS of all animals which excludes the involvement of the vagal pathway in the DON-related information conveyed from the gut to the brain, at least in our experimental model and at the DON doses used. This puzzling result pointed out the existence of an alternative, non characterized mechanism supporting the anorexigenic action of the toxin. We hypothesized a possible action on specific central structures of the orally administered toxin having reached the brain through circumventricular organs. The DON-induced c-Fos staining observed in circumventricular organs and surrounding structures (AP, NTS, ME, ARC) supported this hypothesis. Moreover, DON was shown to be rapidly distributed in various organs including the brain within a short time after exposure [35]. We bring here the first evidence showing that centrally injected DON, at doses ineffective at the periphery, was able to dose-dependently reproduce anorexia observed after *per os* intoxication. DON affected meal pattern in a way similar to that previously observed after peripheral administration i.e. significant decrease of meal frequency and size and parallel increase in intermeal intervals and satiety ratio. Interestingly, the pattern of activated structures was similar after *per os* and icv administration and central DON injection also resulted in the activation of anorexigenic networks including POMC and nesfatin–1 expressing neurons both in the NTS and hypothalamus. The mechanism by which DON activates neuronal networks remains however to be clarified but its action seems faster than would be expected from its inhibitory effect on protein synthesis. DON has been reported to modulate the immune system in murine models and to increase cytokine production in both *in vitro* and *in vivo* murine models (see [2] for review). It is likely that pro-inflammatory mediators could partake in the anorexigenic response to the toxin. In accordance, we recently reported that *per os* DON administration induced an up-regulation of IL-1β, IL-6 and TNF-α mRNA within central structures involved in food intake control [36]. The induction of such pro-inflammatory cytokines within the brain may explain the DON-induced anorexia observed here. A similar increased cytokine expression was indeed observed during anorexigenic immune challenge [37].

In summary, the present work provides the first demonstration that DON modifies feeding behavior by interfering with central neuronal networks dedicated to food intake regulation. Moreover, our results, with particular attention to icv DON injection, strongly suggest that DON reaches the brain where it modifies anorexigenic circuitry activity. These results were obtained with an animal model using acute intoxication with relatively high DON doses. Accordingly, extrapolation on human health should be made with caution. However, we believe these new results are sufficient to reconsider the impact of DON on human population chronically exposed to low toxin doses. The consequences of a possible central energy balance modulation should be evaluated, especially on vulnerable and predisposed individuals suffering from unwanted body weight loss (such as cachexia) or eating disorders (such as anorexia nervosa). Using animal models, future investigations will in particular determine whether chronic consumption of low DON doses worsens such eating disorders. Finally, these points should be addressed in the future for DON alone and consumed in combination with other toxins that may be present in human food.

## Materials and Methods

### Animal housing

Experiments were performed on adult male DBA/1lac J mice of 20–25 g body weight (Janvier). All animals were individually housed in a pathogen-free facility at controlled temperature on a 12/12-h light/dark cycle (lights on at 7 am) with standard powder diet (AO4 P2.5, SAFE UAR) and water available *ad libitum*. Individual cages were designed in order to limit spillage [38]. Mice had free access to standard powder diet *via* two holes made at the bottom of the cage. For habituation, animals were housed in these cages at least 10 days before experiments. All experiments were performed at 21°C. Additional experiments were performed using male proopiomelanocortin (POMC)-Tau-Topaz GFP transgenic mice developed by Pinto and collaborators [39] using the BAC transgenic technology.

### Ethics Statement

Experiments carried out in this study were performed in strict accordance with European Economic Community guidelines (86/609/EEC) and the local committees' recommendations (C-13-055-6, Aix-Marseille University) for the care and use of laboratory animals.

### *Per os* administration of DON

One hour prior to the beginning of the dark phase, mice were administered orally 6.25 to 25 mg/kg body weight (bw) DON (D-0156, Sigma Chemical Co.), dissolved in 100 µl per 10 g body weight of distilled water via gavage, using a 22 gauge intubation needle (Popper and Sons). Prior to DON treatment, mice received the same volume of distilled water using the similar oral administration procedure for a habituation period of seven consecutive days.

### Surgery and intracerebroventricular injection of DON

#### Cannula implantation

Animals were anaesthetized by an intraperitoneal injection of ketamine (100 mg/kg; Imalgen 1000, Merial) and xylazine (6 mg/kg; Rompun, Bayer), and placed in a digital stereotaxic apparatus (Model 502600, WPI) coupled to the neurostar software (Neurostar GmbH). A 26-gauge stainless steel cannula was implanted into the lateral ventricle at the following coordinates: 0.3 mm posterior to bregma, 1.1 mm lateral to the midline, and 2.6 mm ventral to the skull surface. The cannula was secured to the skull with dental cement and sealed with removable obturators. The animals were sutured, placed in individual cages and allowed to recover for seven days. During this resting period, animals were injected with physiological saline every other day for habituation. One week post-surgery, mice were administered either 10 µl (2 µl/min) of physiological saline or DON solution at the beginning of the dark phase. The correct cannula positioning was checked for each animal at the end of the experiment by cresyl violet staining of brain sections. Subgroups of mice were anaesthetized as previously described and fixed with paraformadehyde 4% one hour after injections for immunohistochemistry procedures.

### Food intake Measurements

#### Powdered food consumption

One hour before lights off, mice received either intra-esophageal or icv administration of DON or vehicle. Immediately after treatment, a fresh supply of pre-weighed food was given. The measurement of powdered food intake was the same as in previous studies [33]. Food intake was calculated as the difference between the pre-weighed and the remaining powder measured with a precision balance (0.01 g; Denver Instrument from Bioblock).

#### Continuous food intake measurements and meal pattern analysis

To measure food intake in mice, we customized commercially available polypropylene cages (Cage S; Charles River Laboratories) with a feeding station allowing access to a food jar, placed below the cage, and filled with powdered food (powder AO4; SAFE UAR). The feeding station was adapted from Kurokawa and colleagues [40] and designed in such a manner that neither feces nor urine could drop onto the food jar and that food spillage was reduced to a negligible amount. To analyze meal pattern, the food jar was placed on a precision balance (Ohaus Scout Pro; 200 g range; 0.01 g precision) connected to a computer with a USB/RS232 interface. Custom software, developed with Delphi, allowed the multiplexed continuous data acquisition from 1 to 8 cages. Time series of food jar weight data consisted in stepwise reductions with high amplitude and high frequency oscillations, separated by stabilized values, with low amplitude infrequent oscillations. Analysis first proceeded through a Matlab developed software, to filter out high frequency oscillations in food jar weights and collect the starting time, end time and weight amplitude of the stepwise reductions in food jar weight. Analyzed data were exported to Excel for further analysis. Meals were defined as stepwise reductions in food jar weight of at least 0.02 g in amplitude (i.e. twice the balance precision) and 30 seconds in duration.

#### Measurement of pica behavior (kaolin intake)

Kaolin pellets were prepared from pharmacological grade kaolin and gum Arabic (Sigma Chemical Co) mixed at a 99∶1 ratio in distilled water. The kaolin paste was rolled and cut into pieces similar in shape to mice chow pellets. The pellets were dried in an oven at 37°C for 72 h and then placed into individual cages. Mice were allowed access to regular food and kaolin pellets during a 5 days adaptation period before the beginning of the study. On the day of the experiment the last 24 h kaolin consumption was recorded. Mice were then administered DON (12.5 mg/kg bw) *per os* as described above and kaolin intake was measured daily for the four following days by subtracting preweighed and remaining kaolin pellets with a precision balance (Denver Instrument from Bioblock).

### Vagotomy Surgeries

Animals were anaesthetized with an ip injection of ketamine (100 mg/kg; Imalgen 1000, Merial,) and xylazine (6 mg/kg; Rompun, Bayer), and unilateral cervical vagotomies (UCV) were performed according to previously established procedures [41]. The left vagus nerve, which transfers the majority of gastrointestinal signals to the CNS, was severed. In sham-operated animals, the carotid trunk was exposed but the vagus nerve was not touched. Verification of vagotomy was performed by injecting into the peritoneal cavity a retrograde neuronal tracer, which is transported to cell columns of the DMNX. Seven days before DON administration and sacrifice, animals were given 0.5 ml intraperitoneal injections of 0.4% Fluorogold (# FP46766A, Interchim). Fluorogold staining was observed with a Nikon Eclipse E600 light microscope and images were acquired using a DXM 1200 camera (Nikon) coupled to ACT-1 software. The absence of Fluorogold staining in the left DMNX attested of the correct sectioning of the nerve.

### Quantitative RT-PCR analysis

Animals used for RT-PCR analysis were not refed after DON administration and were sacrificed 3 h after treatment. mRNA expression within the brainstem and the hypothalamus was quantified as described previously [33]. Briefly, total RNA was extracted from frozen hypothalamus using RNeasy Mini kit and RNeasy Micro Kit (Qiagen) respectively. After RNA reverse transcription, gene expression analysis by real time PCR was performed using the ABI Thermocycler 7500 fast (Applied Biosystems). The equivalent of 6.25 ng initial RNA was subjected to PCR amplification in a 10 µl final volume using specific 2.4 µM primers and SYBR Green PCR master mix (Applied Biosystems). Product formation (primers in Table 2) was detected at 60°C in the fluorescein isothiocyanate channel. The generation of specific PCR products was confirmed by melting-curve analysis. For each PCR, cDNA were run in duplicate in parallel with serial dilutions of a cDNA mixture tested for each primer pair to generate a standard linear curve, which was used to estimate relative quantities of gene of interest and of β-actin (internal reference gene) mRNA.

**Table 2:** Primers for SYBR Green assays.

Gene product	RefSeq	Primers	
		Fw	Rv
**AgRP**	NM_007427.2	AGCTTTGGCGGAGGTGCT	GCGACGCGGAGAACGA
**CART**	NM_013732.6	GCCAAGGCGGCAACTTC	TCTTGCAACGCTTCGATCTG
**MC4-R**	NM_016977.3	AAGCTGCCCAGATACAACTTATGA	ACGCGCTCCAGTACCATAACA
**NPY**	NM_023456.2	TCCGCTCTGCGACACTACAT	TGCTTTCCTTCATTAAGAGGTCTG
**POMC**	NM_008895.3	TGAACATCTTTGTCCCCAGAGA	TGCAGAGGCAAACAAGATTGG

### Immunohistochemistry procedures

As mentioned for PCR analysis, *per os* treated-animals used for immunostaining procedure were sacrificed 3 h after treatment without free access to food. Animal perfusion was achieved with 10 ml of 0.1 M PBS followed by 50 ml of 4% paraformaldehyde (PFA) in 0.1 M PBS. Brains were post-fixed for 1 h in 4% PFA at room temperature, rinsed in PBS and then cryoprotected for 24–48 h in 30% sucrose at 4°C. After freezing of the brains in isopentane (−40°C), coronal sections (40 µm thick) were cut on a cryostat (Leica CM3050, France) and collected serially in PBS (0.1 M; pH 7.4). Brains were cut from caudal brainstem (Bregma−8.24 mm) to forebrain (Bregma+0.75 mm).

#### c-Fos immunohistochemistry

c-Fos immunohistochemistry was performed on free-floating sections using an anti-c-Fos rabbit antiserum synthesized against amino acids 4–17 of human protein (1∶10000, Ab-5, Calbiochem) as previously described [41]. Briefly, the free-floating sections were incubated 10 min in a solution containing 0.3% H_2_O_2_ in PBS 0.1 M for quenching of endogenous peroxidase activity. After 1 h in PBS containing 3% normal goat serum (NGS) and 0.3% Triton X-100, sections were incubated for 48 h at 4°C in PBS containing 3% NGS, 0.3% Triton X-100 and anti-c-Fos antibody. A biotinylated goat anti-rabbit IgG (1∶400, Vector Labs) was used as secondary antibody. After incubation with the avidin-biotin complex (1∶200, Vector Labs), horseradish peroxydase activity was visualized using a nickel-enhanced diaminobenzidine (DAB) as the chromogen. The reaction was closely monitored and terminated when optimum intensity was achieved (3–5 min) by washing the sections in distilled water. Non-specific labeling was assessed on alternate slices that were treated identically to the above but in which the primary antibody was omitted. Alternatively, when associated with phenotyping labeling, c-Fos staining was performed using an anti-c-Fos goat IgG (1∶5000; sc-52-G, Santa Cruz) and revealed by means of a donkey-Alexa 594 conjugated anti-goat IgG (1∶400, A11058, Invitrogen). In these cases, the sections were incubated 2 h at room temperature with the secondary antibody.

#### Phenotypic identification

To determine potential activation of POMC, nesfatin-1 and TH expressing neurons within the hypothalamus and brainstem in response to DON (12.5 mg/kg) administration, we performed double immunohistochemistry labeling on c-Fos stained sections. For POMC detection, we used mice expressing the fluorescent GFP under the promoter of POMC gene. GFP was labeled using an anti-GFP rabbit primary antibody (1∶1000, A11122, Molecular Probes). Similarly, identification of nesfatin-1 neurons was performed by incubating sections with a rabbit primary antibody raised against nesfatin-1 (1∶10000, H-003-22-B, Phoenix Pharmaceuticals Inc.). For TH detection purpose, brainstem sections were incubated with an anti-TH rabbit antibody (1∶1000, MAB318, Chemicon). Each labeling was revealed using an Alexa-488-conjugated secondary antibody (1∶400, A11034, Molecular Probes). Finally, all sections were mounted on gelatin-coated slides, air dried, and coverslipped with mounting medium for fluorescence microscope preparation (DAKO).

### Microscopy, image analysis and cell count

c-Fos immunostaining was further analyzed by counting the positive nuclei on four sections. c-Fos positive nuclei counting was performed on photomicrographs acquired using a 10 fold lens with a DXM 1200 Camera (Nikon) coupled to ACT-1 software. The microscope was set at a specific illumination level, as was the camera exposure time. c-Fos positive nuclei were then counted on these pictures by computer-assisted morphometry using the NIH image J software. Images were normalized by subtracting the background determined for each structures studied. The c-Fos stained elements were identified by setting a threshold value (140 grey levels above the background on a 0–255 intensity scale). Counts were manually corrected for overlapping cell nuclei that were counted by the software as single. The software-generated counts of c-Fos stained profiles were also manually corrected by excluding positive objects whose surface area did not exceed 10 pixels (image resolution 150 pixels/inch) corresponding to objects with a surface equal or inferior to 3 square µm. For double labeling, fluorescent images were first acquired on a confocal microscope (Leica TCS SP2) using the 488-nm band of an Ar-laser and the 543-nm band of a He/Ne-laser. Images were sequentially acquired to check marker co-localization. Then, images were routinely acquired with a DXM 1200 Camera (Nikon) coupled to ACT-1 software. Fluorescent images acquired at 520 and 594 nm were merged with phase contrast images to allow visualization of activated neurons. For each brain structure studied and each staining, the counts performed on 4 distinct sections from both hemispheres were averaged.

### Statistical analysis

Data are represented as mean ± S.E.M. Comparisons between data from vehicle- and DON-treated mice were performed using unpaired 2-tailed Student's *t*-test. One-way ANOVA were performed for comparison between data from mice treated with different doses of DON. Tukey's HSD (Honestly Significant Difference) test was used for post hoc analysis. *P* values less than 0.05 were considered significant. The correlations between the kaolin and chow intakes were calculated by chi-square correlation test.
